# COVID-19 Vaccine Reactogenicity Marks an Innate Inflammatory Response Associated With HLA Variation and Enhanced Protection

**DOI:** 10.21203/rs.3.rs-10085252/v1

**Published:** 2026-06-26

**Authors:** Anshika Srivastava, Demetra S.M. Chatzileontiadou, Anurag Adhikari, Rayo Suseno, Sean Lin, Juliano Boquett, Jamie Tuibeo, Tasneem Yusufali, Noah D. Peyser, Ticiana D.J. Farias, Katherine M. Kichula, Andrea T. Nguyen, Irvin Jose, Dhilshan Jayasinghe, Samuel Liwei Leong, Alexis Beatty, Katerina Tarassi, Elissavet Kontou, Janesha C. Maddumage, Kleio Ampelakiotou, Alexandra Tsirogianni, Michael J. Dewar-Oldis, Tuong-Khanh My Tu, Thanh Kha Phan, Peter J. Barnard, Joseph J. Sabatino, Dimitrios Zoulas, Emily Ariens, Timothy R. Mercer, Emma J. Grant, Lloyd D’Orsogna, Corey Smith, Paul J. Norman, Gregory M. Marcus, Jeffrey E. Olgin, Mark J. Pletcher, Martin Maiers, Stephanie Gras, Jill A. Hollenbach

**Affiliations:** 1. Department of Neurology, University of California San Francisco, San Francisco, CA 94158, USA; 2. Immunity and Infection Program, La Trobe Institute for Molecular Science, La Trobe University, Bundoora, VIC 3086, Australia; 3. Department of Biochemistry and Chemistry, School of Agriculture, Biomedicine and Environment, La Trobe University, Bundoora, VIC 3086, Australia; 4. Department of Biochemistry and Molecular Biology, Monash University, Clayton, VIC 3800, Australia; 5. Department of Infection and Immunology, Kathmandu Research Institute for Biological Sciences, Lalitpur, Nepal; 6. Department of Medicine, University of California San Francisco, San Francisco, CA 94158, USA; 7. University of Colorado Anschutz School of Medicine, Aurora, CO; 8. Immunology-Histocompatibility Department, “Evangelismos” General Hospital, Athens, Greece; 9. Blood Bank Department, “Evangelismos” General Hospital, Athens, Greece; 10. Research Centre for Extracellular Vesicles, La Trobe Institute for Molecular Science, La Trobe University, Melbourne, VIC 3086, Australia; 11. BASE mRNA Facility, Australian Institute for Bioengineering and Nanotechnology (AIBN), University of Queensland, Brisbane, QLD 4067, Australia; 12. School of Medicine, University of Western Australia, Nedlands 6009, Australia; 13. Department of Clinical Immunology and PathWest, Fiona Stanley Hospital, Murdoch 6150, Australia; 14. Queensland Immunology Research Centre and Inflammation and Infection Program, QIMR Berghofer Medical Research Institute, Brisbane, QLD 4006, Australia; 15. School of Biological Sciences, University of Queensland, Brisbane, QLD 4072, Australia; 16. Department of Epidemiology and Biostatistics, University of California San Francisco, San Francisco, CA 94158, USA; 17. Division of General Internal Medicine, University of California San Francisco, San Francisco, CA 94158, USA; 18. CIBMTR (Center for International Blood and Marrow Transplant Research), NMDP, Minneapolis, MN 55401-1206, USA

## Abstract

Vaccination against SARS-CoV-2 has been central to mitigating the COVID-19 pandemic, although transient systemic side effects remain common and contribute to vaccine hesitancy. While previous studies have implicated *HLA* variation in COVID-19 vaccine reactogenicity, the mechanisms linking immunogenetic variation, inflammatory side effects, and protection remain poorly understood. Here, using a large deeply genotyped and phenotyped cohort, together with functional immunological analyses, we investigated the determinants of vaccine reactogenicity. In 50,535 vaccinated individuals, with replication in an independent cohort of 4,575 individuals, we confirmed a remarkably strong association between HLA-A*03:01 and systemic side effects following COVID-19 vaccination (OR = 1.36, CI = 1.31–1.41, p = 6.79 × 10^−57^). In contrast, HLA-B*08:01 was associated with reduced vaccine reactogenicity, that extended across both COVID-19 and influenza vaccines, suggesting separable antigen-specific and generalized determinants of vaccine reactogenicity. HLA-A*03:01 carriage was additionally associated with fewer breakthrough and recurrent SARS-CoV-2 infections, while individuals reporting stronger vaccine side effects exhibited reduced infection risk and milder disease course independent of *HLA* genotype. History of allergy was likewise associated with increased vaccine reactogenicity, consistent with a broader host predisposition to inflammatory responsiveness. Unexpectedly, despite the strong *HLA* association, we did not observe evidence of enhanced antigen-specific T-cell activation in HLA-A*03:01^+^ individuals. Instead, our immunological analyses pointed toward a prominent role for early inflammatory cytokine responses from monocytes, with inflammatory signatures correlating with vaccine side-effect severity selectively among HLA-A*03:01^+^ donors. Together, these findings support a model in which vaccine reactogenicity reflects the interaction of antigen-specific immunogenetic effects and broader innate inflammatory responsiveness. More broadly, our results suggest that transient vaccine side effects may represent a clinically observable correlate of protective immune activation.

## Introduction

COVID-19 vaccines are an important public health tool in preventing severe illness, hospitalization, and mortality due to infection with the virus(1–4). In early studies examining the efficacy of these vaccines, mRNA vaccines BNT162b2 (referred to hereafter by the brand name “Pfizer”) and mRNA-1273 (referred to hereafter by the brand name “Moderna”) were shown to be 95% and 94.1% effective against symptomatic COVID-19 infection, respectively(5). Adenovirus-based vaccines, such as ChAdOx1-S (brand name “Johnson & Johnson”, hereafter “J&J”) and Ad26.COV2.S (brand name “AstraZeneca”), in contrast, showed 70% and 66% efficacy, respectively(6, 7). Thus, although mostly efficacious in preventing severe illness and hospitalization, ‘breakthrough’ infections (BTI) can occur after vaccination, independently of vaccine brands. These infections may be attributed to vaccine failure, secondary vaccine failure, individuals’ age, immune evasion by novel viral variants, or waning vaccine efficacy over time(8–15).

Prior work has indicated that the immunogenicity of the COVID-19 vaccines can vary by individual and is correlated with their efficacy(16). For example, studies have reported variable immunological responses to vaccines linked to age, sex, body mass index, nutritional status, and the composition of the gut microbiome(17, 18). Along with these factors, neutralizing antibody titers and binding antibody levels have emerged as key immune correlates of protection for COVID-19 vaccines(19, 20). Suggesting an immunogenetic feature of vaccine response, several studies have linked variation in the human leukocyte antigen (*HLA*) region with antibody levels and T-cell responses after vaccination(21–24). *HLA* is the most polymorphic region (6p21) of the human genome with tens of thousands of known alleles. Variation in *HLA* is long recognized to play a role in viral illness(25). Demonstrating the importance of HLA antigen presentation in the immune response to SARS-CoV-2, we have previously identified a specific *HLA* allele that is associated with an asymptomatic disease course(26) and provided a functional and structural basis to explain the association. Likewise, numerous other *HLA* associations with the COVID-19 disease course have been identified(27).

While overwhelmingly safe, vaccination-induced immune activation can lead to vaccine reactogenicity, often referred to as “side effects.” Most side effects are mild, including fever, muscle aches, headaches, and fatigue, as well as local reactions such as pain, redness, and swelling at the injection site(28). These mild reactions typically appear within a few hours after vaccination and are short-lived, usually resolving within one to two days for most individuals(29). Although rare adverse events such as myocarditis have been reported following mRNA vaccination and are hypothesized to involve some *HLA* alleles, their underlying mechanisms remain poorly understood(30). While bothersome, transient vaccine reactogenicity has previously been linked to innate immune activation associated with stronger vaccine-induced immune responses in influenza vaccination studies(31), although this relationship remains incompletely characterized for COVID-19 vaccines^32^. A study involving participants receiving the human papillomavirus vaccine, for example, found that the occurrence of inflammation-related adverse reactions is associated with blood antibodies levels, suggesting that individuals who have vaccine-induced side effects have a more robust humoral immune response(32, 33). Importantly, systemic side effects from COVID-19 vaccination, such as fever and fatigue, have also been associated with enhanced humoral and cellular immune responses(34). Previous work has shown a specific *HLA* class I allele, HLA-A*03:01 to be associated with increased COVID-19 vaccine reactogenicity and increased antibody response(22, 35, 36), as well as enhanced memory T cells after COVID-19 mRNA booster doses(23), primarily with mRNA vaccines. This leaves open the question of whether such associations are specific to the mRNA platform.

In addition, the biological basis of vaccine reactogenicity remains poorly understood, and it is unclear whether such associations are unique to COVID-19 vaccines or reflect more generalized predisposition to inflammatory vaccine responses.

Here, we investigate the epidemiology of COVID-19 vaccine reactogenicity in a large cohort of more than 50,000 vaccinated individuals with high-resolution *HLA* genotyping. Integrating genetic association analyses with functional immune profiling, we identify distinct patterns of vaccine-associated immunogenetic effects. We observed that vaccine reactogenicity association of HLA-A*03:01 was COVID-19 vaccine-specific, whereas a broader protective effect of HLA-B*08:01 was observed across both COVID-19 and influenza vaccines. Interestingly, carriage of HLA-A*03:01 was also linked to a reduced incidence of breakthrough and recurrent SARS-CoV-2 infections. Despite these clear *HLA* associations, we did not detect robust antigen-specific T-cell responses in HLA-A*03:01^+^ individuals. Instead, our results suggest an important contribution of early monocyte-derived inflammatory cytokines, highlighting that transient vaccine side effects mark an innate inflammatory response that may represent a clinically observable correlate of protective immune activation. This work sheds light on the mechanisms underlying *HLA*-mediated COVID-19 vaccine reactogenicity and associated reduction in infections, providing important new insights that may support efforts to optimize vaccine efficacy and promote broader public involvement in vaccination programs.

## Results

### Participant recruitment and baseline demographics

Between January 2023 and January 2024, we collected responses to survey questions regarding respondents’ general health history, including experiences with COVID-19 and associated vaccinations, from potential bone marrow donors registered with the NMDP (formerly National Marrow Donor Program/Be The Match) and for whom high-resolution *HLA* genotyping data were available in the NMDP database. The specific survey questions related to COVID-19 and vaccinations, including side effects queries, are given in **Supplementary Table 1**. Because this was a U.S.-based cohort, respondents received only vaccines approved for use in the country (Pfizer, Moderna, J&J). Among the 80,007 total respondents, 50,535 self-identified as White (**Supplementary Table 2a**) and reported having completed at a minimum the initial series of vaccination for SARS-CoV-2 (one dose for J&J, two for Pfizer/Moderna mRNA); these subjects constituted our discovery cohort. An additional 7,403 respondents reported completion of the initial series self-identified with other ancestries (**Supplementary Table 2b**).

For our replication cohort, we collected responses between July 2020 and April 2022 via a mobile phone app with follow-up daily questions specific to vaccine side effects, as described in Augusto et al., 2023(26). Among 10,595 respondents who reported completion of the initial vaccination series, 4,575 self-identified as White (**Supplementary Table 3**). While these respondents are also NMDP donors with available high resolution *HLA* data, there was no overlap with individuals in the discovery cohort.

### Systemic side effects of COVID-19 vaccines co-occur and are associated with HLA-A*03:01

To understand the distribution of side effects to COVID-19 vaccines in our discovery cohort, we first calculated the covariation matrix for all reported side effects (**Supplementary Figure 1**). Here, we only considered side effects reported after completion of the initial vaccination series. We found that mild systemic side effects (SSE) like fever or chills, muscle or body fatigue, or headaches, rather than localized side effects like runny nose (**Supplementary Table 4**), showed substantial co-occurrence, with a median number of two SSE reported per individual.

**Figure 1 |:**
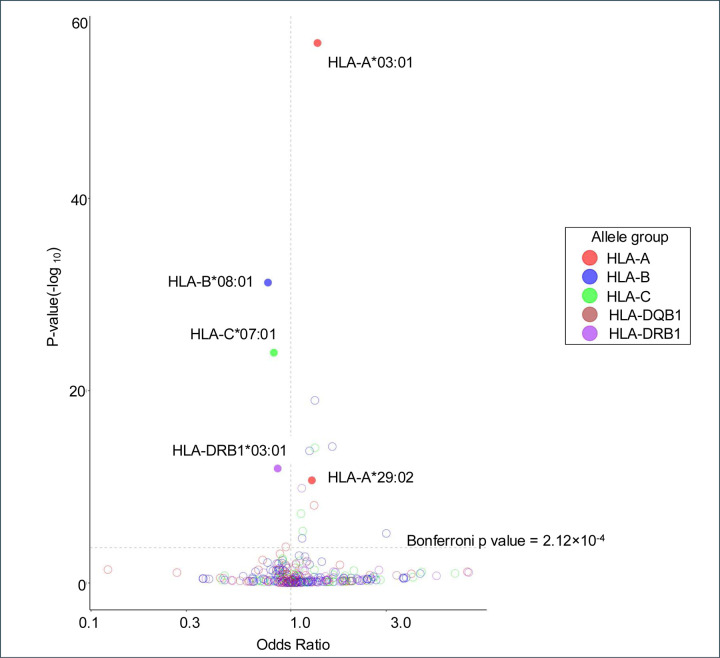
Association of *HLA* alleles with three or more systemic side effects in the discovery cohort (Self-identified White, N = 50,535). Odds ratio is shown on the x-axis and p-value is shown on the y-axis. *HLA* alleles that are significant in both discovery and replication cohort are shown as solid-colored circles.

To evaluate whether *HLA* variation plays a role in increasing SSE in vaccination for SARS-CoV-2 and to capture cases with the greatest burden of symptoms, we first considered individuals who reported greater than the median number of SSE. Using a dominant model, we used multivariate logistic regression to test for association with the occurrence of three or four reported SSE for each *HLA* allele at each classical class I (*HLA-A, -B, -C*) and two *HLA* class II (*HLA-DRB1* and *-DQB1*) loci observed at a frequency >3% in our cohort, adjusting for sex and age (Figure 1, **Supplementary Table 5**). This revealed as the top candidate a strong and significant association of HLA-A*03:01 with SSE (OR = 1.36, CI= 1.31 – 1.41, p = 6.79×10^−57^). We did not observe any substantial dose effect for HLA-A*03:01 (homozygous, OR = 1.47, CI = 1.29 – 1.68, p = 1.65×10-^8^; heterozygous, OR = 1.40, CI = 1.34 – 1.46, p = 3.15×10-^53^), confirming the dominant model. Because HLA-A*03:01 belongs to the HLA-A3 supertype group(37) that also includes HLA-A*11:01, -A*31:01, -A*33:01, and -A*68:01, and is characterized by shared peptide binding motif(38), we hypothesized that this supertype might show shared associations with SSE across alleles. However, we found that among the *HLA-A3* supertype alleles, only HLA-A*03:01 was significant for the association with increased SSE (**Supplementary Table 5**). This association of increased SSE with HLA-A*03:01 clearly replicated in our replication cohort of 4,575 vaccinees who self-identified as White, with a remarkably consistent effect size (OR = 1.46, CI = 1.21 – 1.77, p = 7.77 × 10^−5^) relative to that in our discovery cohort (**Supplementary Table 6)**. In addition, we found that HLA-A*03:01 was significantly associated with increased SSE reports in our self-identified Hispanic cohort (N = 4,287), again with extremely consistent effect size (OR = 1.45, CI = 1.25 – 1.69, p = 7.31×10^−7^, **Supplementary Table 7**). While a similar trend was clearly observed, this association did not reach statistical significance in cohorts of other ancestries, where our sample sizes were much smaller (**Supplementary Tables 8** and **9**). Therefore, we performed a meta-analysis to evaluate this association across ancestry groups. Effect estimates were broadly consistent, with odds ratios (ORs) ranging from 1.24 to 1.46. The strongest association was observed in the Hispanic cohort (OR = 1.46, 95% CI: 1.26–1.69), followed by the White cohort (OR = 1.36, 95% CI: 1.31–1.41), whereas estimates in the Black cohort (OR = 1.29, 95% CI: 0.90–1.85) and the Asian or Pacific Islander cohort (OR = 1.24, 95% CI: 0.85–1.80) were directionally consistent. The White cohort contributed most of the weight (92.1%) (**Supplementary Figure 2**). Under a common-effect model, the pooled OR was 1.36 (95% CI: 1.32–1.42; z = 16.71, p <1×10^−4^), indicating strong evidence for association of HLA-A*03:01 with COVID-19 vaccine reactogenicity. There was no evidence of heterogeneity across studies (Q = 1.13, d.f. = 3, p = 0.77), with negligible between-study variance (τ^2^ = 0; I^2^ = 0%).

To better understand whether any specific reported side effects was driving this HLA association, we considered the association with HLA-A*03:01 for each side effect separately (**Supplementary Table 10**). As expected, we observed a strong and highly significant negative association of HLA-A*03:01 with reporting “no vaccine side effects” (OR = 0.73, CI = 0.70 – 0.77, p = 5.82×10^−43^), demonstrating that individuals with this allele are less likely to report having experienced no side effects after vaccination. Analysis of reports of specific side effects revealed the strongest association of HLA-A*03:01 with “fever or chills” (OR = 1.43, CI = 1.38 – 1.49, p = 1.37×10^−81^), followed by “muscle or body aches” (OR = 1.305, CI = 1.25 – 1.35, p = 2.16×10-^45^), “fatigue” (OR = 1.25, CI = 1.20 – 1.30, p = 5.57×10^−34^), and “headaches” (OR = 1.23, CI = 1.19 – 1.29, p = 1.00×10^−24^). We also found remarkably consistent results for our cohort of individuals who self-identify as Hispanic (**Supplementary Table 10**, **Supplementary Figure 3**). Only “fatigue” was found to have a significant association with HLA-A*03:01 in our Black cohort (OR = 1.49, CI = 1.07 – 2.09, p = 0.018); however, as noted previously, smaller sample sizes limited our power to detect associations in some cohorts (**Supplementary Tables 8** and **9**). Finally, we did not find a substantive difference in effect size when considering individuals who had reported infection prior to vaccination or breakthrough infection (BTI) (**Supplementary Table 11**).

In summary, we find a highly significant, consistent association of HLA-A*03:01 with reported side effects post-COVID-19 vaccination. This association replicated across multiple large independent cohorts, ancestries and vaccine platforms, supporting a role for *HLA* variation driving vaccination reactogenicity in a broad and global manner.

### HLA-A*03:01 is associated with vaccine reactogenicity across vaccine brands, with varying effect size

Because previous studies have primarily linked HLA*03:01-driven reactogenicity to a single platform (Pfizer)(35), we sought to determine whether association with other vaccine platforms could be detected in our larger cohort. We similarly found that while the median number of SSE reported across the entire discovery cohort was two, the proportion of individuals who experienced three or four SSE was higher in Moderna than in Pfizer or Johnson & Johnson (J&J) vaccinated individuals (**Supplementary Figure 4**). Because the frequency of SSE varied by vaccine manufacturer, we stratified our cohorts according to whether they received the Pfizer, Moderna, or J&J vaccine. To reduce confounding, we only considered individuals who received both doses of the initial series (in the case of mRNA vaccines) from the same manufacturer.

We confirmed that Pfizer-vaccinated individuals showed the largest effect size for all associations of HLA-A*03:01 with side effects relative to other vaccine brands, particularly fever or chills (OR = 1.71, CI = 1.61 – 1.79, p = 4.51×10-^88^). However, the chance of not experiencing any side effects when carrying HLA-A*03:01 is significantly lower in both Pfizer (OR = 0.676, CI = 0.64 – 0.71, p = 2.83×10^−40^) and Moderna recipients (OR = 0.782, CI = 0.72 – 0.85, p =1.59×10^−9^) compared to J&J (**Supplementary Table 12**). While we did not observe significant associations of HLA-A*03:01 with most side effects among J&J vaccine recipients, likely owing to the small sample size for this vaccine brand (N = 3,312), we did find a clear significant effect with respect to fatigue (OR = 1.259, CI = 1.09 – 1.45, p = 1.40×10^−4^) and a strong trend for other SSE. This demonstrates that the observed *HLA* association is not specific to the mRNA platform. We observed similar results in our replication cohort, where the association of HLA-A*03:01 showed similar patterns of effect size between brands (i.e. Moderna and Pfizer), although some individual side effects did not reach statistical significance among Moderna recipients, likely due to more limited power in this cohort; nevertheless, detectable associations across vaccine platforms suggest that the role of HLA-A*03:01 in mediating vaccine reactogenicity is relatively uniform across vaccine platforms (**Supplementary Table 13**).

Thus, we find that HLA-A*03:01 is associated with vaccine reactogenicity across COVID-19 vaccine brands, although this association is much more pronounced and consistent among Pfizer recipients.

### Additional HLA class I alleles are associated with SSE in COVID-19 vaccination

In addition to HLA-A*03:01, we discovered numerous other *HLA* alleles that were significantly associated with reports of SSE in our cohorts (Figure 1, **Supplementary Table 5**). Of these, HLA-A*29:02 (OR = 1.28, CI = 1.19 – 1.36, p = 2.10×10-^11^), HLA-B*08:01 (OR = 0.76, CI = 0.73 – 0.80, p = 5.65×10^−32^), HLA-C*07:01 (OR = 0.82, CI = 0.79 – 0.85, p = 1.10×10-^24^), and HLA-DRB1*03:01 (OR = 0.85, CI = 0.82–0.90, p = 1.23×10^−12^) were clearly replicated (**Supplementary Table 6**). HLA-B*08:01, HLA-C*07:01, and HLA-DRB1*03:01 are components of the well-documented “ancestral 8.1 haplotype” (AH8.1)(39), which is also evident from the high linkage disequilibrium (LD) values between these alleles (**Supplementary Table 14**). Owing to the likelihood that these three alleles reflected a single primary association through LD, we performed conditional analyses to identify the primary associated allele in this haplotype. We found that HLA-B*08:01 had the strongest effect size (OR = 0.77, CI = 0.71 – 0.84, p = 2.71×10^−9^) after controlling for HLA-C*07:01, and HLA-DRB1*03:01, suggesting that HLA-B*08:01 allele is responsible for the protective effect against SSE. Likely owing to the small sample size, we did not detect these associations in our cohorts with other ancestries (**Supplementary Tables 7 – 9**).

In summary, while HLA-A*03:01 demonstrated the strongest and most significant association with respect to vaccine SSE, we find evidence for additional *HLA* class I association with both risk (HLA-A*29:02) and protection (HLA-B*08:01) from COVID-19 vaccine side effects. The identification of these additional *HLA*-associated SSE expands upon prior work, which largely implicated HLA-A*03:01 alone and did not describe broader *HLA* class I contributions to vaccine reactogenicity. Taken together, these results support a broader role for HLA-mediated antigen presentation in shaping vaccine reactogenicity and suggest that individual immunogenetic background may influence both the magnitude and quality of post-vaccination immune responses.

### HLA-A*03:01 associated vaccine reactogenicity is specific to COVID-19 vaccines, while HLA-B*08:01 is globally protective

To distinguish COVID-19-specific from generalized determinants of vaccine reactogenicity, we next examined SSE associated with seasonal influenza vaccination in the same individuals. This analysis provided an opportunity to determine whether the observed *HLA* associations reflected mechanisms unique to COVID-19 vaccination or broader host predisposition to inflammatory vaccine responses. We administered a follow-up questionnaire to the same individuals included in our discovery cohort (**Supplementary Table 15**). This survey specifically assessed the occurrence of influenza vaccine-associated side effects and the frequency with which participants received influenza vaccinations. As of April 2025, we received responses from a total of 13,499 participants from our discovery cohort, among whom 11,916 individuals reported having received the influenza vaccine at least once (**Supplementary Table 16**). Of those receiving the vaccine, 3,417 individuals reported experiencing side effects. We performed multivariate logistic regression to assess associations between each *HLA* allele and the presence of SSE (**Supplementary Table 17**). Here, we chose the presence of at least one SSE as the primary outcome, as the number of reported SSE for the influenza vaccine was lower than that for the COVID-19 vaccines, with a median of one side effect reported.

Strikingly, we found that the strong association between HLA-A*03:01 and vaccine reactogenicity was not observed for influenza vaccination, supporting a COVID-19 vaccine-specific effect of this allele. In contrast, HLA-B*08:01 demonstrated a consistent negative association with SSE across both COVID-19 and influenza vaccines. Among the alleles tested (HLA-A*03:01, HLA-A*29:02, and HLA-B*08:01) only HLA-B*08:01 demonstrated a significant association (OR = 0.83, CI = 0.72–0.95, p = 0.0095) with influenza vaccine SSE at p < 0.05. These findings suggest that distinct immunogenetic mechanisms contribute to vaccine reactogenicity, including both antigen-specific effects and broader host determinants of inflammatory responsiveness.

Importantly, despite the absence of shared *HLA* risk alleles across vaccines, individuals reporting SSE following influenza vaccination were more than three times as likely to report SSE following COVID-19 vaccination, even after adjustment for *HLA* genotype (OR = 3.09, CI = 2.73–3.50, p = 2 10^−16^). Together, these findings support the existence of at least two separable contributors to vaccine reactogenicity: antigen- or platform-specific immunogenetic effects, exemplified by HLA-A*03:01, and broader host inflammatory predisposition that transcends individual vaccines.

### Individuals reporting vaccine SSE also report fewer breakthrough infections, fewer total infections, and milder disease course

Owing to the highly significant association of HLA-A*03:01 with vaccine side effects, we next asked whether this allele might be associated with reduced breakthrough infection (BTI). Of the total vaccinated 57,938 individuals across ancestries, we excluded 10,227 individuals who did not have information for their 2^nd^ dose timing. Among the remaining participants who reported specific dates for both vaccination and infection, we found HLA-A*03:01 to be associated with a decreased risk of BTI, albeit with modest effect size (OR = 0.916, CI = 0.88 – 0.95, p = 1.61×10^−5,^). This association was also significant with stronger effect size in our cohort with Hispanic ancestry (OR = 0.776, CI = 0.66 – 0.91, p = 1.44×10^−4^), and while not reaching statistical significance, we observed similar effect sizes in other ancestries (**Supplementary Table 18**). We did not observe any other significant *HLA* associations with BTI in our cohort, including previously reported associations of HLA-DQB1*06(21, 40).

The observed association of HLA-A*03:01 with reduction in BTI is also reflected in an overall decrease in total infections reported. We dichotomized participants according to those reporting having never been infected or having only a single infection, (about 86% of our discovery cohort, N= 43,527), and those reporting repeated reinfections (i.e. two or more infections, about 14% of our discovery cohort, N = 7,008) between 2019 and 2023. We found that HLA-A*03:01^+^ vaccinated individuals were less likely to have experienced repeated SARS-CoV-2 infections (OR = 0.92, CI = 0.87 – 0.96, p = 0.00177). Finally, we asked whether among those who did report infection, HLA-A*03:01 is associated with milder disease course. We found that HLA-A*03:01^+^ individuals were less likely to report higher than the median number (three) of symptoms (considering all reported symptoms; OR = 0.90, CI = 0.88 – 0.94, p = 2.78×10^−7^). Additionally, severe COVID-19 vaccine reactogenicity remained inversely associated with COVID-19 symptom burden (OR = 0.80, CI = 0.77 – 0.84, p = 2.0×10^−16^) even after adjustment for HLA-A*03:01, indicating that individuals experiencing stronger vaccine SSE tended to have a milder disease course irrespective of *HLA* status.

To understand whether the HLA-A*03:01 association with reduced BTI was secondary to its association with side effects, we considered the occurrence of vaccination side effects and the likelihood of BTI irrespective of *HLA* genotype. We found that each side effect was independently inversely associated with the likelihood of a BTI (**Supplementary Table 19**). Considering the combined measure of three or four SSE and BTI (and adjusting for sex, age, and vaccine brand), we found a highly significant protective effect with an effect size greater than that observed for HLA-A*03:01 (OR = 0.83, CI = 0.79 – 0.87, p = 6.03×10^−14^). When we include HLA-A*03:01 as a covariate in our model, we observed a nearly identical association of SSE with BTI (OR = 0.82, CI = 0.79 – 0.87, p = 9.40×10^−14^), while the HLA-A*03:01 (OR = 0.93, CI = 0.89 – 0.98, p = 5.81×10^−3^) association was only borderline significant, suggesting an association independent from *HLA*. Finally, we stratified our cohort according to the carriage of HLA-A*03:01 and further observed a highly significant and consistent inverse association between the combined measure for three or four SSE and the risk of BTI in individuals without the HLA-A*03:01 allele (OR = 0.81, CI = 0.78 – 0.85, p = 4.32×10^−19^).

Thus, we conclude that the negative association of HLA-A*03:01 with BTI stems from its role in increasing vaccine reactogenicity, along with a global negative association of vaccine SSE with BTI.

### Vaccine reactogenicity is associated with history of allergy independent of HLA

To understand the epidemiology of vaccine reactogenicity, we next sought to further investigate the role of individuals’ health histories in mediating side effects. Respondents in our discovery cohort reported broadly on their health histories, allowing us to examine the association of a range of phenotypes with vaccine reactogenicity(41). After controlling for age, sex, HLA-B*08:01 and HLA-A*03:01, individuals with a history of allergies exhibited an increased likelihood of reporting most SSE following both COVID-19 and influenza vaccinations (**Supplementary tables 20** and **21**, respectively). The direction of effect was consistent across vaccine types, suggesting that allergy status may be associated with increased vaccine reactogenicity in a virus and platform independent manner. No other phenotype, including autoimmune and other immune-mediated conditions, was associated with vaccine reactogenicity in this cohort. Importantly, the association was consistent across diverse allergy phenotypes with differing immunological bases, including classical IgE-mediated allergy, delayed hypersensitivity reactions, and nonclassical chemical sensitivities. This concordance across mechanistically distinct allergic conditions is consistent with the notion that vaccine reactogenicity reflects, at least in part, variation in shared innate pathways independent of HLA-restricted adaptive immunity.

### HLA-A*03:01 carriage is associated with higher antibody levels after COVID-19 vaccination

We next sought to understand the immune mechanism that drives the HLA-A*03:01 association with COVID-19 vaccine SSE. We first confirm prior reports of association of HLA-A*03:01 with higher levels of anti-SARS-CoV-2 antibody after COVID-19 vaccine (22, 42, 43) in an independent cohort of 156 healthcare workers receiving two doses of the Pfizer COVID-19 vaccine. The cohort was stratified in three groups according to the levels of anti-Spike IgG antibodies after the second dose of the vaccine as follows, Group I: 1,000 – 4,000 AU/mL (N = 50), Group II: 4,001 – 20,000 AU/mL (N = 53), Group III: ≥ 20,000 AU/mL (N = 53). To investigate the association between HLA-A*03:01 and anti-Spike IgG levels, we performed logistic regression comparing Group III against Groups I and II. Our results confirmed that HLA-A*03:01 is associated with higher anti-Spike IgG levels post vaccination (OR = 3.25, CI = 1.31 – 8.33, p = 0.0117). We also observed that anti-Spike IgG antibody positivity increased with HLA-A*03:01 allele dosage (0, 1, or 2 copies; **Supplementary Figure 5**). Thus, we confirm that in addition to its role in mediating vaccine SSE, individuals positive for HLA-A*03:01 display a higher anti-Spike IgG antibody response post-vaccine, providing further evidence that vaccine SSE are reflective of a robust humoral immune response to COVID-19 vaccination and that this has an immunogenetic underpinning.

### Spike-derived HLA-A*03:01 dominant KCY-specific T cell activation is modest

**Figure 2 |:**
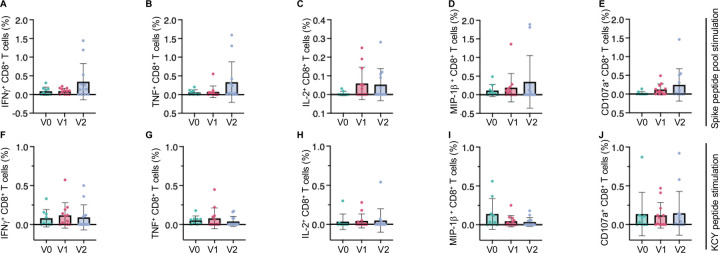
Modest Spike-specific CD8^+^ T cell responses after COVID-19 vaccination in HLA-A*03:01^+^ samples. (**A-E**) Cell lines generated by stimulating PBMCs with the Spike peptide pool and restimulated with the Spike peptide pool. Frequencies of IFNγ^+^, TNF^+^, IL-2^+^, MIP-1β^+^ and CD107a^+^ CD8^+^ T cells from HLA-A*03:01^+^ samples at baseline (V0, n = 9), after one (V1, n = 12) or two (V2, n = 12) vaccine doses. (**F-J**) Cell lines generated by stimulating PBMCs with the Spike peptide pool and restimulated with the KCY peptide. Frequencies of IFNγ^+^, TNF^+^, IL-2^+^, MIP-1β^+^ and CD107a^+^ CD8^+^ T cells from HLA-A*03:01^+^ samples at baseline (V0, n = 9), after one (V1, n = 12) or two (V2, n = 12) vaccine dose. Bars show mean ± SD and symbols denote individual donors. Data represent biological replicates from independent donors (n) with one technical replicate per condition. Statistical comparisons were performed using a two-sided Mann–Whitney U test and were all not significant.

To understand the COVID-19 vaccine reactogenicity association with HLA-A*03:01, we next assessed the T cell response towards Spike from HLA-A*03:01^+^ donors. We compared the T cell response to Spike-derived peptides before (V0, n = 9) and after one (V1, n = 12) or two vaccine doses (V2, n = 12) (**Supplementary Table 22**). T cell lines were generated with Spike-derived peptide pools covering the entire protein and restimulated with the peptide pools (Figure 2A–E, **Supplementary Figures 6–12**). CD8^+^ and CD4^+^ T cell responses were modest and independent of the vaccine dosage, similar to previous findings(23). Next, we focused on the response to the known dominant HLA-A*03:01-restricted Spike-derived epitope _378_KCYGVSPTK_386_, hereafter named KCY(23, 44). T cell lines were generated with the Spike-derived peptide pools and restimulated with the KCY peptide in samples collected before and after vaccination (Figure 2F–J, **Supplementary Figures 8–10**). Limited IFNγ production was observed in ~50% of the samples before vaccination (V0, n=4/9, 0.08 ± 0.11 %), and a slight increase in IFNγ production was observed after vaccination (mean: V1: 0.12 ± 0.16 %, V2: 0.09 ± 0.16 %) (Figure 2F, **Supplementary Figures 8–10**). Similarly, the production of the other effector functions tested (TNF, IL2, MIP-1β and CD107a) was low before and after vaccination towards the KCY peptide (Figure 2G–J).

The KCY-specific CD8^+^ T cell response was modest compared with other well characterized Spike-derived epitopes such as the HLA-A*02:01-restricted S_269_-_277_ epitope (mean of 5.85% IFNγ^+^ CD8^+^ T cells(45)) or the HLA-B*15:01-restricted S_919_-_927_ epitope (mean of 0.36% IFNγ^+^ CD8^+^ T cells in pre-pandemic samples(26)). We compared the T cell response towards KCY to a previously characterized HLA-A*03:01-restricted influenza-derived peptide, Flu-NP_265_(46), in the same samples (n = 6). The T cell response to the Flu-NP_265_ peptide was substantially stronger than that to the KCY peptide, with IFNγ production being ~13-fold higher (mean ± SD; Flu-NP_265_: 3.32 ± 2.36 %; KCY: 0.27 ± 0.19 %) (**Supplementary Figure 13**), TNF production ~25-fold higher (mean ± SD; Flu-NP_265_: 3.31 ± 2.28 %; KCY: 0.13 ± 0.06 %), and CD107a ~10-fold higher than KCY (mean ± SD; Flu-NP_265_: 3.32 ± 2.36 %; KCY: 0.34 ± 0.33 %) (**Supplementary Figure 13**). The response towards the Flu-NP_265_ peptide demonstrates that HLA-A*03:01^+^ CD8^+^ T cells can produce high level of cytokines, and the relatively weak response observed toward KCY is specific, rather than reflecting globally poor CD8^+^ T cell response in HLA-A*03:01^+^ donors.

In summary, the CD8^+^ T cell response towards the Spike-derived peptides, even towards the KCY epitope, was modest after COVID-19 vaccine, and therefore T cell activation is unlikely to underpin the SSE observed in HLA-A*03:01^+^ individuals.

### High frequency of na ve KCY-specific tetramer^low^ T cell population is observed ex vivo

**Figure 3 |:**
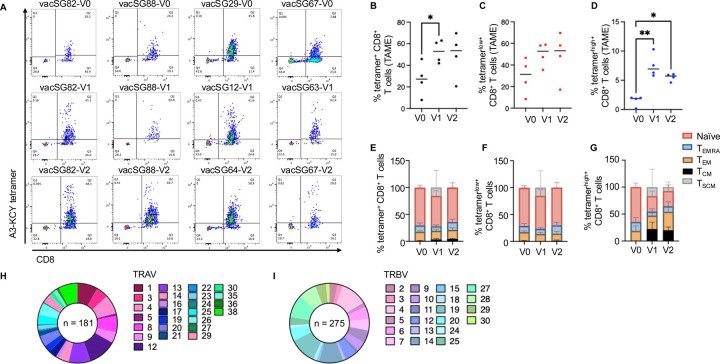
Tetramer^low^ KCY-specific CD8^+^ T cells are present before vaccination and tetramer^high^ population emerges after vaccination. (**A**) Tetramer-associated magnetic enrichment (TAME) plots of HLA-A*03:01-KCY-specific CD8^+^ T cells across before (V0, n = 4) and after vaccination (V1, V2, n = 4, respectively). (**B**) Quantification of KCY^+^ CD8^+^ T cell frequencies by TAME. (**C-D**) Mean fluorescence intensity (MFI) analysis of HLA-A*03:01-KCY-specific CD8^+^ T cells with (**C**) tetramer^low^ and (**D**) tetramer^high^ at V0, V1 and V2. (**E-G**) Phenotypic composition of (**E**) all tetramer^+^, (**F**) tetramer^low^ and (**G**) tetramer^high^ HLA-A*03:01-KCY-specific CD8^+^ T cells showing the proportion of naïve (red), effector memory (T_EM_, orange), central memory (T_CM_, black), stem like memory cells (T_SCM_, grey), and terminally exhausted memory re-expressing RA cells (T_EMRA_, blue) phenotype. Bars show mean – SD and symbols denote individual donors. Data represent biological replicates from independent donors (n) with one technical replicate per condition, unmatched samples. *p < 0.05; **p < 0.01. Statistical comparisons were performed using a two-sided Mann–Whitney U test. (**H-I**) KCY-specific TCR clonotypes with the (**H**) the TCR a-chain sequences colored by TRAV (TCR a variable gene) and (**I**) the TCR b-chain sequences colored by TRBV (TCR b variable gene). Each TCR gene segment is shown in a different color.

It has been shown that in COVID-19 recovered individuals KCY-specific memory T cells increase upon vaccination(23). Therefore, we wanted to determine if the same applies without prior infection. We performed *ex vivo* tetramer-associated magnetic enrichment (TAME) to analyze the phenotype of the KCY^+^ CD8^+^ T cells in samples collected before and after vaccination (Figure 3, **Supplementary Figure 14**). Surprisingly, even prior to vaccination (V0) a high proportion of KCY^+^ CD8^+^ T cells was present in all samples (Figure 3A). The proportion of KCY^+^ CD8^+^ T cells increased after vaccination (Figure 3A–B), and a distinct population of KCY^+^ T cells with high mean fluorescence intensity (MFI) emerged (tetramer^high^) and increased after vaccination compared to baseline (mean tetramer^high+^ at V0: 1.49 ± 0.89 %; V1: 7.49 ± 2.01 %; V2: 5.53 ± 0.65 %) (Figure 3D).

We next assessed whether the phenotype of KCY^+^ T cells *ex vivo* was different between the tetramer^high^ and the tetramer^low^ populations (Figure 3E–G, **Supplementary Figure 15**). The majority of the tetramer^low^ T cells exhibited a naïve phenotype (T_N_: CCR7^+^/CD45RA^+^) independent of the vaccination status (mean of 69.95, 56.35 and 63.9 % for V0, V1 and V2, respectively), and some stem cell memory T cells (T_SCM_: CCR7^+^/CD45RA^+^/CD95^+^) present especially in one V1 sample (Figure 3F, **Supplementary Figure 15**). The tetramer^low^ effector memory T cell (T_EM_: CCR7-/CD45RA-) frequency was ~14%, and the terminally differentiated effector T cell (T_EMRA_: CCR7-/CD45RA^+^) frequency was around ~12% independent of vaccination status. In contrast, the tetramer^high^ T cell phenotypes changed upon vaccination (Figure 3G). The tetramer^high^ naïve T cell proportion decreased after vaccination (V0: 64.7%, V1 28.4%: V2: 28.0%), while effector (V0: 18.6%, V1: 24.9% V2: 34.2%) and central (V0: 0.0%, V1: 22.2% V2: 19.9%) memory T cell proportion increased (Figure 3G, **Supplementary Figure 15**). In two samples collected > 400 days after the 3^rd^ vaccine dose (vacSG64-V5 and vacSG86-V5), we observed 36% and 8.85% of KCY^+^ T_EMRA_ cells, respectively, and surprisingly, a large population of naïve cells (32% and 79.7%, respectively) (**Supplementary Figure 15B**). In addition, the large proportion of low MFI tetramer^+^ T cells, even after vaccination (Figure 3E) was specific to the KCY peptide and not observed for another well characterized spike-derived peptide S_269_ restricted to HLA-A*02:01 (**Supplementary Figure 16**). We further confirmed the peptide specificity of the tetramer^+^ T cells by performing co-staining with both tetramers of KCY and Flu-NP_265_ epitopes presented by the HLA-A*03:01 molecule (**Supplementary Figure 17**).

Finally, we determined the T cell receptor (TCR) repertoire specific to the KCY peptide. Using single-cell sorting of tetramer^+^CD8^+^ T cells and TCR sequencing, we obtained 324 productive clonotypic sequences with 95 paired αβTCRs from eight donors before and after vaccination (**Supplementary Table 23**). The TCR repertoire was private with no biased TCR gene usage observed, and more surprisingly no clonotypes were preferentially expanded (Figure 3H–I). We only observed a few persisting clonotypes that were maintained before and after vaccination and/or after different vaccine doses in different donors, and a public TCR α chain shared between two donors (**Supplementary Table 24**).

Overall, these TCR repertoire findings suggest a broader, polyclonal T cell response rather than the expansion of dominant clonotypes, consistent with a modest T cell response characterized by a predominantly naïve phenotype.

### Full-length Spike protein stimulated high expression of IL-6 and IL-8 by monocytes

Despite the high number of tetramer^+^ KCY-specific T cells present (Figure 3), we did not observe an enhanced T cell activation that could explain vaccine reactogenicity associated with HLA-A*03:01 carriage, as hypothesized in previous studies(35). Therefore, we asked whether other immune cells could lead to inflammation that would underpin mild vaccine side effects associated with HLA-A*03:01 carriage. To address this, we stimulated PBMCs with the different vaccine components: the empty lipid nanoparticle (LNP) using the Pfizer vaccine formula(47), the soluble full-length Spike protein (HexaPro)(48), or the Spike-derived peptide pools (S1 and S2); as well as positive and negative controls.

**Figure 4 |:**
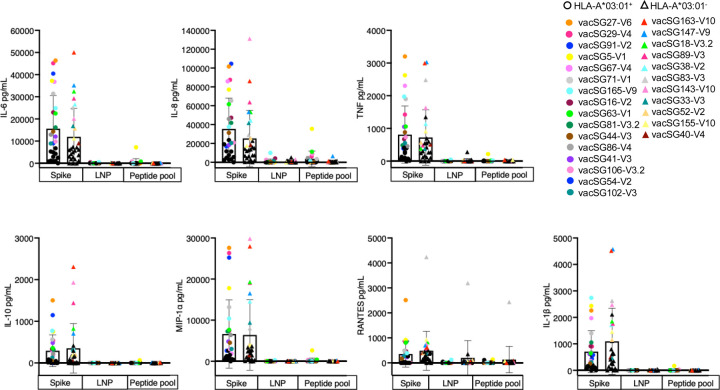
Whole Spike protein stimulation activates large IL-6 and IL-8 production Cytokine levels measured in PBMCs from HLA-A*03:01^+^ (n = 27, dots) and HLA-A*03:01- (n = 28, triangles) samples after stimulation with full-length soluble Spike protein (Spike), lipid nanoparticle (LNP), or Spike peptide pool (Peptide pool). Only full-length Spike induced broad cytokine responses, including IL-6, IL-8, TNF, IL-10, MIP-1α, RANTES, IL-1β. Bars represent mean – SD; symbols denote individual samples, colored dots or triangles represent the samples with high cytokine level production while the samples with low cytokine level production are in black. Data represent biological replicates from independent donors (n) with one technical replicate per condition. Statistical comparisons were performed using a two-sided Mann–Whitney U test and were all not significant.

Among the 55 samples tested (n=27 HLA-A*03:01^+^ and n=28 HLA-A*03:01-, **Supplementary Table 22**) high cytokine production was mainly observed after stimulation with the full-length soluble Spike protein (Figure 4, **Supplementary Figure 18**). As there was variability of response between the samples tested, we focused on the secreted cytokines for which the average concentration was well above baseline(49). Cytokines strongly induced by the whole Spike protein stimulation included Interleukin (IL)-6, IL-8, IL-10, IL-1β, tumor necrosis factor (TNF), macrophage inflammatory protein-1alpha (MIP1α), and RANTES (Figure 4). In addition, Interferon-γ (IFNγ), granulocyte-macrophage colony-stimulating factor (GM-CSF), inducible protein 10 kDa (IP-10), monocyte chemoattractant protein-1 (MCP-1), and IL-1a were also produced upon whole Spike protein stimulation, but at lower concentrations (**Supplementary Figure 18**). The increase of cytokine production was observed in both HLA-A*03:01^+^ and HLA-A*03:01- samples, with no significant difference between the two groups. However, IL-6 and IL-8 were the only cytokines to show a notable elevated production in HLA-A*03:01^+^ samples after Spike stimulation representing ~1.3- and ~1.4-fold increase compared with HLA-A*03:01-, respectively (Figure 4). IL-6 and IL-8 cytokines were also expressed at the highest concentration compared to other cytokines.

**Figure 5 |:**
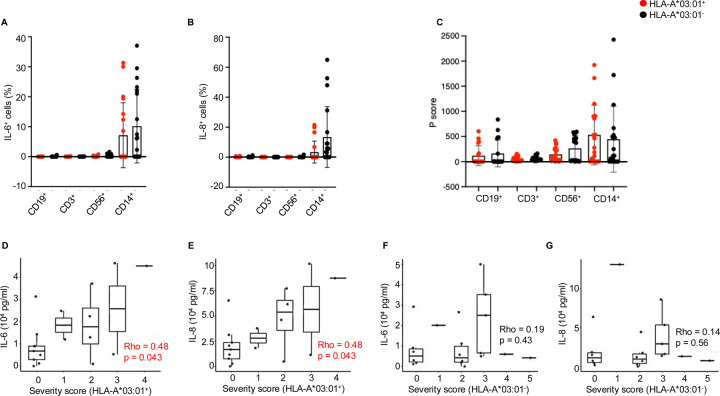
IL-6 and IL-8 cytokines are mainly produced by monocytes, and their levels correlate with vaccine side-effect severity for HLA-A*03:01^+^ samples. (**A-B**) Intracellular cytokine staining of Spike-stimulated PBMCs showing IL-6^+^ (**A**) and IL-8^+^ (**B**) cells among CD3^+^ T cells, CD19^+^ B cells, CD56^+^ NK cells, and CD14^+^ monocytes from HLA-A*03:01^+^ (red dots, n = 19) and HLA-A*03:01- samples (black dots, n = 19). (**C**) Phagocytic score (P score) representing the uptake of Spike-coated microbeads by monocytes, NK, T and B cells in HLA-A*03:01^+^ (red dots) and HLA-A*03:01- samples (black dots). Bars represent mean – SD; symbols denote individual donors. (**D-G**) Correlation of IL-6 (**D**, **F**) and IL-8 (**E**, **G**) concentrations with self-reported vaccine side-effect severity scores from HLA-A*03:01^+^ (**D-E**, n=18, dots) and HLA-A*03:01- donors (**F-G**, n=20, dots). Data represent biological replicates from independent donors (n) with one technical replicate per condition. Correlations were evaluated using two-sided Spearman’s rank correlation coefficient (r).

To determine which cell subset was responsible for the production of IL-6 and IL-8, cytokine production was assessed in Spike-stimulated PBMCs, with and without Spike stimulation, using flow cytometry. IL-6 was produced predominantly by monocytes (CD14^+^) despite the low number of CD14^+^ cells in blood (Figure 5A, **Supplementary Figures 19** and **20A-B**, **Supplementary Table 22**), and at lower levels by natural killer (NK) cells (CD56^+^), suggesting a primary role of the innate response in vaccine reactogenicity (Figure 5A). In HLA-A*03:01^+^ samples (n = 19), IL-6^+^ NK cells averaged 0.14 ± 0.2 % and monocytes 7.2 ± 10.8 % (n = 7/19), whereas in HLA-A*03:01- samples (n = 19) comparable NK responses (0.29 – 0.44 %) but broader monocyte positivity (10.2 – 12.3 %, n = 11/19) were observed (Figure 5A). In contrast, IL-6^+^ T and B cells were rare, with only traceable responses (< 0.05 %). Similarly, IL-8 was produced by monocytes, averaging 3.4 – 7.3 % (n = 4/19) in HLA-A*03:01^+^ and 13.4 ± 20.3 % (n = 11/19) in HLA-A*03:01- samples, while other subsets were negligible (Figure 5B). In contrast with the high level of IL-6 and IL-8 secreted (Figure 4), the frequencies of IL-6^+^ and IL-8^+^ cells detected were low in PBMCs. Therefore, we performed a Spike uptake assay in PBMCs, further confirming that Spike uptake was mainly driven by monocytes showing a higher phagocytic score, and at lower level by NK cells (phagocytosis of Spike-coated microbeads; Figure 5C, **Supplementary Figure 20C**).

In summary, the high levels of IL-6 and IL-8 production were observed only in the presence of the full Spike protein and were primarily produced by monocytes.

### Expression of IL-6 and IL-8 correlates with side effect severity in HLA-A*03:01 donors

While our results suggest a role for IL-6 and IL-8 in vaccine reactogenicity, the differences observed between HLA-A*03:01^+^ and HLA-A*03:01- samples were not significant. However, as with most complex phenotypes, the association of HLA-A*03:01^+^ with vaccine reactogenicity is incompletely penetrant. While more HLA-A*03:01^+^ individuals report SSE with vaccination, there was a range of severity reported among our PBMC donors; thus, we sought to determine whether levels of IL-6 and IL-8 were correlated with reported vaccine side effect severity in these donors. Strikingly, in HLA-A*03:01^+^ samples (n = 18, **Supplementary Table 25**), both IL-8 and IL-6 levels showed strong and significant positive correlations with side effect severity score (IL-8 and IL-6: rho = 0.48 and p = 0.043, Figure 5D–E), indicating that higher cytokine production was associated with increased vaccine side effect severity. In contrast, no significant correlation was observed in HLA-A*03:01- individuals (n = 20, **Supplementary Table 25**, Figure 5F–G). To ensure that the correlation observed in HLA-A*03:01^+^ samples was due to the transient presence of the Spike protein, we checked the baseline levels of IL-6 and IL-8 cytokines in serum of HLA-A*03:01^+^ donors collected prior to vaccination, or two weeks post-first and -second vaccine dose, alongside TNF and IFNγ as controls (**Supplementary Figure 21**). Overall, the level of cytokines was low (< 100 pg/mL) or moderate, and no significant increase was observed before or after vaccination. This demonstrates that the cytokine production upon Spike presentation is likely transient.

Together, these data establish that HLA-A*03:01^+^ samples display a distinct pro-inflammatory signature, with IL-6 and IL-8 production strongly linked to vaccine side-effect severity. The early and transient nature of this cytokine production primarily by monocytes strongly suggests an innate immune response underlying HLA-A*03:01 vaccine reactogenicity.

## Discussion

Vaccination has been a crucial public health intervention for decades, significantly contributing to the prevention and control of infectious diseases worldwide. However, concerns about vaccine safety and efficacy, and negative public perceptions regarding side effects, have emerged as a significant challenge in maintaining high vaccination coverage. Because the binding between HLA and peptide antigen is highly specific and a fundamental component in initiating the adaptive immune system, understanding the role of *HLA* variation in vaccine response can be crucial in determining factors that underlie vaccine efficacy. Variation in HLA-A*03:01 has previously been reported as associated with COVID-19 vaccine reactogenicity(35, 36, 40). Here, in a substantially larger cohort than previously examined, we clarify and extend these observations and place them into a broader immunogenetic framework. Our findings suggest that vaccine reactogenicity reflects the interaction of antigen-specific *HLA* effects and more generalized determinants of inflammatory responsiveness, and that these responses are linked to protection from subsequent infection.

Leveraging a large, registry-based cohort of more than 50,000 individuals, we expand understanding of *HLA* variation as a major determinant of COVID-19 vaccine reactogenicity, with HLA-A*03:01 and HLA-A*29:02 associated with increased risk of SSE and HLA-B*08:01 associated with protection. Interestingly, we discover that HLA-A*03:01 carriage was associated not only with increased COVID-19 vaccine reactogenicity, but also with reduced risk of BTI, fewer total SARS-CoV-2 infections, and a milder disease course. Notably, the association of vaccine reactogenicity with better protection (less BTI and milder disease) extended beyond HLA-A*03:01 itself, suggesting that SSE represent a broad correlate of effective immune activation upon vaccination. While the HLA-A*03:01 association appeared specific to COVID-19 vaccination, the SSE inverse correlation with HLA-B*08:01 extended across both COVID-19 and influenza vaccines. Together, these findings suggest that vaccine reactogenicity reflects the interaction of antigen-specific immunogenetic effects and broader host determinants of inflammatory responsiveness.

Owing to the crucial role of HLA class I molecules in antigen presentation, the role of CD8^+^ T cells in HLA-A*03:01-mediated vaccine reactogenicity presented a clear initial line of inquiry into the mechanisms underlying reported SSE. We observed a high frequency of CD8^+^ T cells capable of recognizing the immunodominant Spike-derived peptide restricted by HLA-A*03:01, KCY specifically(23), even prior to vaccination or infection. Interestingly, both prior and subsequent to vaccination, the majority of the CD8^+^ T cells exhibited low mean florescence intensity (tetramer^low^) and had a naïve phenotype. Post-COVID-19 vaccination we did observe the expansion of tetramer^high^ KCY-specific CD8^+^ T cells with a memory phenotype. However, in contrast with HLA-A*02:01^+^ samples, even after multiple doses of the COVID-19 vaccine, the frequency of KCY^+^ naïve CD8^+^ T cells remained high, persisted with time, likely reflecting the large proportion of naïve T cells able to specifically bind the KCY peptide independent of vaccine status. Most strikingly, even post-vaccination, the T cell response to KCY was muted relative to responses to an Influenza-derived peptide, such that it is unlikely that the observed COVID-19 vaccine reactogenicity can be attributed to an enhanced T cell activation. Consistent with the modest antigen-specific T-cell responses observed, it is also possible that the distinctive polyclonal TCR repertoire associated with HLA-A*03:01 limits early antigen clearance, prolonging antigen availability and thereby increasing the contribution of innate and cytokine-driven responses to the overall vaccine response.

In line with this, our data point toward innate inflammatory pathways as a central determinant of HLA-associated reactogenicity. Monocytes emerged as the predominant source of IL-6 and IL-8 following Spike stimulation, and the magnitude of these cytokine responses correlated directly with vaccine side-effect severity in HLA-A*03:01^+^ donors. Together with our epidemiologic findings linking reactogenicity across vaccine types and allergy phenotypes, these results suggest that HLA-A*03:01-associated reactogenicity reflects an amplified innate inflammatory response rather than exaggerated antigen-specific T-cell activation. Notably, IL-6 is a key regulator of T follicular helper cell differentiation, germinal center responses, and antibody production(50), providing a plausible mechanistic link between the increased reactogenicity associated with HLA-A*03:01 and the enhanced antibody responses observed here and in previous studies(43). While the precise causal pathway remains to be established, our findings support a model in which transient innate inflammatory activation contributes both to post-vaccination SSE and to the development of more effective humoral immunity, consistent with effective immune activation with enhanced protection from infections (less BTI) and reduced disease severity.

This study is intrinsically constrained by its dependence on self-reported data to assess transient mild vaccine side effects in both the discovery and replication cohorts, which potentially can lead to some imprecision in association results. Additionally, sample size limitations restrict some significant findings to individuals who self-identify as White. Likewise, our cohort was predominantly female (78.4%), which may limit the generalizability to broader populations. Moreover, the low number of monocyte cells in the blood limited the ability to show significant differences, in addition to the incomplete penetrance of the observed genetic effect, which is typical for complex traits. The transient features of SSE also constrained these observations.

Despite these limitations, our findings provide important new insight into the biological basis of COVID-19 vaccine reactogenicity. By integrating large-scale immunogenetic and epidemiological analyses with functional immune studies, we show that vaccine side effects reflect the interaction of antigen-specific *HLA* effects and broader innate inflammatory responsiveness, and that these responses are associated with protection from subsequent infection and disease severity. More broadly, these results suggest that transient vaccine SSE may represent a clinically observable correlate of effective immune activation, providing a framework for understanding individual variation in vaccine responses and informing efforts to optimize vaccine efficacy and public participation in vaccination programs.

## Methods

### Discovery cohort

We recruited our study population via email to all potential volunteer bone marrow donors registered in the NMDP database with available email addresses and high-resolution HLA genotyping information available. The email contained a custom link directing them to a consent page for a health history survey. Subject recruitment, consent process, and survey administration were conducted using both email outreach and a web interface to ensure effective data collection. Upon consenting to provide responses and allow linking with their HLA genotype data, participants spent ten to fifteen minutes completing a detailed survey to gather baseline information and health history.

As of January 19th, 2024, a total of 80,016 eligible donors completed the survey. Among these respondents, 667 individuals (0.83%) were excluded for filling out the survey multiple times, while 1,301 participants were removed due to incomplete HLA variation data. After excluding these cases and individuals that were also participated in the study that formed our replication cohort, below (N = 836), there remained a total of 77,212 participants in the study. Of these individuals, 56,938 people who self-identified as White, Hispanic, Black, or Asian or Pacific Islander, had completed their initial series of vaccinations. We excluded participants who identified as multiple ancestry, unknown ancestry or Native American due to small sample sizes.

### Replication cohort

Our replication cohort consisted of NMDP participants who also participated in the COVID-19 Citizen Science Study, a mobile app-based longitudinal study built using the Eureka Digital Research Platform (University of California San Francisco) as previously described in detail in Augusto et al. 2023(26). Once enrolled, the participants are asked to complete an initial 10 to 15-minute survey about baseline demographics, their health history, and daily habits. Follow-up daily questions specific to vaccine side effects are delivered by push notification or text message on an ongoing basis and require 5 to 15-minute per week. All the participants provided written informed consent agreeing to the research and publication of research results.

We restricted our analysis to individuals who had self-identified as ‘White’ due to insufficient numbers for analysis in the other groups, allowing an analysis of 10,595 individuals reporting vaccination for SARS-CoV-2. Of those, 4,575 individuals completed their initial series of vaccinations. Symptoms are self-reported at the baseline and in daily surveys. Within the baseline survey, the respondents were asked to report whether they had any of a list of symptoms (**Supplementary Table 26**) for 3 days or longer at any time after their complete dose of vaccination.

### Serum antibody levels in vaccinated subjects

The population examined consisted of 156 healthcare workers from “Evangelismos” General Hospital in Athens, Greece, including doctors, nurses, pharmacists, biologists, dentists, technicians and administrative staff. Enrollment was open to all hospital personnel scheduled for vaccination and not restricted by any pre-specified criteria. All individuals received two doses of the mRNA Pfizer-BioNTech vaccine. Data on prior SARS-CoV-2 infection and symptoms experienced after each dose were collected for all participants. Antibody concentrations were assessed at two time points: 21 – 1 days after the first dose and 24 – 2 days after the second dose. Levels of circulating SARS-CoV-2 anti-Spike IgG (S) and anti-nucleocapsid IgG (N) antibodies were quantified using the Abbott Diagnostics SARS-CoV-2 IgG chemiluminescent microparticle immunoassay (Abbott Diagnostics, Abbott Park, Illinois) on an Abbott Diagnostics Architect i2000 SR and an Alinityi Analyzer, according to the manufacturer’s instructions. Results were expressed in AU/mL and were interpreted as positive if ≥ 50 AU/mL(51). Informed consent was obtained from all participants, and the study was approved by the Institutional Review Board of “Evangelismos” Hospital (PN 9/21-01-21). High resolution HLA class I and II genotyping was performed as described(52).

### Peripheral blood mononuclear cells (PBMCs)

HLA-A*03:01^+^ and HLA-A*03:01- donors vaccinated with the Comirnaty BNT162b2 COVID-19 mRNA vaccine (Pfizer), the Oxford–AstraZeneca COVID-19 vaccine (AstraZeneca), or the mRNA-1273 Spikevax COVID-19 vaccine (Moderna) were recruited (**Supplementary Table 22**). PBMCs were separated from whole blood or buffy coats using density-gradient centrifugation. PBMCs were used fresh or were cryogenically stored until use. All individuals consented to research and publication of research results and had been previously HLA genotyped. Ethics approval to undertake the research was obtained from the La Trobe University Human Research Ethics Committee (HEC21097). The HLA genotyping was performed by AlloSeq Tx17 (CareDx Pty) using AllType NGS high-resolution genotyping on the IonTorrent NGS platform or by the Department of Clinical Immunology and PathWest at Fiona Stanley Hospital, Murdoch, Australia.

### Tetramer-associated magnetic enrichment (TAME)

Peptide-loaded HLA-A*03:01 tetramers were generated using Streptavidin conjugated to phycoerythrin (PE). Tetramer-stained cells were enriched using anti-PE antibody-coated immunomagnetic beads on LS columns (Miltenyi Biotech) according to manufacturer instructions. After enrichment, cells were stained with an antibody panel including anti-CD3-BV480 (dilution 1:100), anti-CD8-PerCP-Cy5.5 (1:50), anti-CD4-FITC (1:100), anti-CD14-APCH7 (1:200), anti-CD19-APCH7 (1:100), anti-CD45RA-BUV395 (1:100), anti-CD27-APC (1:100), anti-CCR7-PE-Cy7 (1:50), anti-CD95-BV421 (1:50), anti-PD1-BV605 (1:100), anti-CXCR5-BV650 (1:100) (all BD Biosciences) and Live/Dead Fixable Near-IR Dead Cell Stain (1:1,000) (Life Technologies). Cells were resuspended in MACS buffer (PBS, 0.5% BSA, 2 mM EDTA) and were analysed using the BD FACSymphony A3 system or and were directly single-cell index sorted into PCR plates (Eppendorf) using the BD FACSAria Fusion system.

For the tetramer staining experiments, the TAME cells were stained for 1 hour at room temperature with the APC-conjugated Flu-NP_265_ HLA-A*03:01-restricted peptide tetramer, followed by surface staining using the same antibody panel as above, excluding anti-CD27-APC. Gating strategy shown on **Supplementary Figure 6**.

### Generation of peptide-specific CD8^+^ T cell lines

CD8^+^ T cell lines were generated as previously described(53, 54). In brief, PBMCs were incubated with 1 μM of individual peptide or 10 μg/mL of Spike-derived peptide Pool 1 (25 μg/peptide, 15mers, 1–126) and Pool 2 (25 μg/peptide, 15mers, 127 – 253) (Mimotopes B#33200); and cultured for 10–14 days in RPMI-1640 supplemented with 2 mM MEM non-essential amino acid solution (Sigma-Aldrich), 100 mM HEPES (Sigma-Aldrich), 2 mM l-glutamine (Sigma-Aldrich), penicillin–streptomycin (Life Technologies), 50 mM 2-ME (Sigma-Aldrich) and 10% heat-inactivated fetal bovine serum (Bovogen). The cultures were supplemented with 10 IU IL-2 2–3 times weekly. CD8^+^ T cell lines were used fresh for subsequent analysis.

### Intracellular cytokine assay

CD8^+^ T cell lines were stimulated with 1 μM of individual peptide or 2 μg/mL of the SARS-CoV-2 Spike-derived peptide Pool 1 (25 μg/peptide, 15mers, 1–126) and Pool 2 (25 μg/peptide, 15mers, 127 – 253) (Mimotopes B#33200) and were incubated for 4 – 5 hour in the presence of GolgiPlug, GolgiStop and anti-CD107a-FITC (dilution 1:100) (all BD Biosciences). After stimulation, cells were surface stained for 30 min with anti-CD3-BV480 (1:100), anti-CD8-PerCP-Cy5.5 (1:50) and anti-CD4-BV650 (1:100) antibodies (all BD Biosciences) and Live/Dead Fixable Near-IR Dead Cell Stain (Life Technologies). Cells were fixed and permeabilized using BD Cytofix/Cytoperm solution (BD Biosciences) and then intracellularly stained with anti-IFN-γ-BV421 (1:100), anti-TNF-PE-Cy7 (1:100), anti-IL2-PE (1:100) and anti-MIP-1β-APC (1:100) antibodies (all BD Biosciences) for a further 30 min. Cells were acquired on the BD FACSymphony A3 system using the FACSDiva software (v.9.0.). Post-acquisition analysis was performed using FlowJo software (v.10). Cytokine detection levels identified in the no-peptide control condition were subtracted from the corresponding test conditions in all summary graphs to account for non-specific, spontaneous cytokine production. Gating strategy shown on **Supplementary Figure 6**.

### Single-cell multiplex PCR

Single-cell multiplex PCR was performed as previously described (26). In brief, cDNA was generated using the VILO cDNA synthesis kit (Invitrogen) at 1/20 of the manufacturer’s recommendations with 0.1% Triton X-100. Nested PCR comprising 40 α- and 27 β-chains was subsequently undertaken. PCR products were purified using ExoSAP (GE Healthcare) and were sequenced at AGRF. Sequences were analysed using FinchTV (Geospiza v.1.5.0) and IMGT software (55). CDR3 sequences shown are all productive (no stop codons).^59^

### PBMCs short-term stimulation

1 × 10^6^ PBMCs were stimulated with either 15 μg/mL of empty Pfizer-BioNTech lipid nanoparticle (LNP)(47) or 15 μg/mL custom made SARS-CoV-2 Spike protein (Wuhan strain); or 7.5 μg/mL of the SARS-CoV-2 Spike-derived peptide Pool 1 (25 μg/peptide, 15mers, 1–126) and 7.5 μg/mL Pool 2 (25 μg/peptide, 15mers, 127 – 253) (MIMOTOPES B#33200); or Cell Stimulation Cocktail (500X) (eBioscience^™^); or nothing (Negative Control) for 15 hours in RPMI-1640 supplemented with 2 mM MEM non-essential amino acid solution (Sigma-Aldrich), 100 mM HEPES (Sigma-Aldrich), 2 mM l-glutamine (Sigma-Aldrich), penicillin–streptomycin (Life Technologies), 50 mM 2-ME (Sigma-Aldrich) and 10% heat-inactivated fetal bovine serum (Bovogen). Following the 15-hour stimulation, the cells were restimulated for a further 2-hour using the same conditions, and the supernatant was collected for the BD Cytometric Bead Array (CBA).

The same conditions were also used in the presence of GolgiPlug and GolgiStop (BD Biosciences). Following the 15-hour stimulation and the 2-hour restimulation, the cells were surface-stained for 30 minutes with anti-CD3-BV480 (1:100), anti-CD14-PerCP-Cy5.5 (1:100), anti-CD16-BV421 (1:50), anti-CD19-APC (1:50), anti-CD56-PECy7 (1:50) antibodies (all BD Biosciences) and Live/Dead Fixable Near-IR Dead Cell Stain (Life Technologies). Cells were fixed and permeabilized using BD Cytofix/Cytoperm solution (BD Biosciences) and then intracellularly stained with anti-IL6-FITC (1:50) and anti-IL8-PE (1:50) antibodies (both BD Biosciences) for a further 30 minutes. Cells were acquired on the BD FACSymphony A3 system using the FACSDiva software (v.9.0.). Gating strategy shown on **Supplementary Figure 19**.

### BD Cytometric Bead Array (CBA)

Following the PBMC short-term stimulation, the supernatant level of Interleukin (IL)-1α, IL-1β, IL-2, IL-4, IL-5, IL-6, IL-7, IL-8, IL-9, IL-10, IL-12p70, IL-13, Interferon (IFN)-α, IFNγ, IFNγ inducible protein 10 kDa (IP-10), granulocyte-macrophage colony-stimulating factor (GM-CSF), Lymphotoxin-alpha (LT-α), Eotaxin, Monocyte Chemoattractant Protein-1 (MCP-1), macrophage inflammatory protein-1 alpha (MIP-1α), RANTES, tumor necrosis factor (TNF) and were measured using the BD Cytometric Bead Array (CBA, BD Biosciences) following the manufacturer’s instructions. Samples were acquired in a BD FACSymphony A3 system using the FACSDiva software (v.9.0.). The analysis was performed using the BD CBA Analysis Software (v1.1.15). Cytokine detection levels identified in the Negative Control condition were subtracted from the corresponding test conditions in all summary graphs to account for non-specific, spontaneous cytokine production.

### SARS-CoV-2 Spike-specific phagocytosis assay

After PBMCs stimulation with full length Spike protein for 15-hours, PBMCs (1×10^5^ cells in 50μL) were added onto 60 μL of the donor plasma opsonised microbeads (10μL plasma and 50μL microbeads), mixed by gentle tapping, adjusted to 600 μL using RPMI 1640 containing 0.1% human serum and 0.1 M HEPES pH 7.4 and transferred into 37”C, 5 % CO_2_ incubator. After 2-hour incubation, cells were washed once with 1mL of cold PBS containing 0.5 % FBS and 0.005 % sodium azide and gentle centrifugation at 335 × g for 5 minutes at 4”C, fixed in 400 μL of 1 % paraformaldehyde, and kept at 4”C in the dark until data acquisition using BD FACSymphony A3. A total of 2 ×10^4^ events per tube were acquired from each condition. Relevant assay controls included cells incubated with no beads, and Spike-coated nonopsonized beads. The frequency of cells that phagocytosed the beads (% of cells that took up the beads) and their fluorescent intensities (amounts of beads taken up per cell) were analyzed using BD FlowJo version 10.5.0 software. Phagocytic scores (p-score) were then calculated based on the frequency of cells that took up the opsonized beads denoting the number of positive cells and mean fluorescence intensity (MFI) representing the average bead uptake by the positive cells as described. A positive p-score was defined as three standard deviations above the background mean phagocytic score of healthy donors as described previously(56).

In selected experiments, the intracellular uptake of the opsonized microbeads by effector cells was confirmed by confocal microscopy as described(56). In brief, PBMCs (1×10^4^ cells) after phagocytosis assay were washed twice with cold PBS containing 0.5% FBS and 0.005 % of sodium azide, fixed with 1% paraformaldehyde for 5 minutes at room temperature, and rinsed twice with PBS. The fixed cells were blocked with 1% BSA in PBS, incubated with Alexa-555-conjugated Phalloidin (1:1000, Sigma, USA) for 30 minutes at room temperature, mounted in DAPI nuclear stain-containing media (Molecular Probes, USA), and imaged using ZEISS LSM 880 confocal microscope (Carl Zeiss AG, Germany); featuring 63X/1.4 Plan-Apochromat Oil Immersion objective, with Diode 405 nm (DAPI), Argon ion 488 nm (Alexa-488) and DPSS 561 nm (Alexa-555 phalloidin) laser excitation sources, emitted light was filtered using a combination of emission filters and imaged onto Airy detector array producing an effective lateral resolution of ~100 nm. All the images were Airyscan processed with Zen Black Edition (Zeiss Software).

### Assessment of vaccine side effects

To standardize the evaluation of vaccine-associated SSE, we developed a composite scoring framework termed the SSE severity score adapted from the vaccine side effect guideline described elsewhere(57). Each reported symptom was first categorized by type and severity (local, systemic, or severe) according to previously established criteria for vaccine reactogenicity. For each donor and vaccine dose, symptoms were graded as: 0 = none; 1 = mild (e.g., injection site pain, mild fatigue, or a single local symptom); 2 = moderate (systemic but not severe, e.g., fever, chills, headache, myalgia, or multiple mild symptoms); or 3 = severe (multiple systemic symptoms, prolonged recovery >2 days, swelling requiring medical review, dose-limiting reaction, or hospitalization). To account for compounded burden, additional multipliers were applied: +1 if multiple symptoms occurred at the same dose, +1 if symptoms recurred across multiple doses, and +1 if symptom duration exceeded 2 days (if reported). Scores were then summed to generate an overall SSE severity score per participant, which was categorized as: 0 (no side effects), 1–2 (mild), 3–4 (moderate), and ≥ 5 (severe). The scores are summarized in **Supplementary Table 25**.

### Statistical analysis

*HLA* associations: In our discovery cohort, we examined the association of five *HLA* loci (*HLA-A, -B, -C, -DRB1, -DQB1*). Data analysis included the first two fields of the allele name as described in the HLA nomenclature, representing the complete molecule at polypeptide sequence resolution. We calculated allele frequencies for all the *HLA* loci, haplotype frequencies using Haplostats R package (58) and R2 for Linkage Disequilibrium (from our in-house script) between all the pair of loci (**Supplementary Table 14**). We employed a generalized logistic regression model using ‘glm’ in the R (V 4.3) base package to consider relevant covariates, including sex and age. For the replication cohort, we tested only the allele of interest, using the generalized logistic regression model framework as described. We utilized our in-house Python script to construct forest plots. We conducted an analysis of variance (ANOVA) to assess the statistical significance of differences in antibody positivity rates across groups with different counts of HLA-A*03:01 allele (0,1 or 2). For meta-analysis to evaluate *HLA* allele association across ancestry groups, we used inverse variance method, with between-study variance estimated via restricted maximum likelihood (REML). Confidence intervals for τ^2^ and τ were derived using the Q-profile method, and I† was calculated based on Cochran’s Q statistic.

All additional analyses were performed in GraphPad Prism v10.1. Data are shown as mean – SD, with symbols representing individual donors. Paired comparisons across time points were assessed using two-tailed Wilcoxon matched-pairs tests, and unpaired comparisons between HLA-A*03:01^+^ and HLA-A*03:01^−^ groups by two-tailed Mann–Whitney U tests. For the CBA, cytokine values were background-subtracted from unstimulated or controls; analytes below detection limits were excluded. Correlations between cytokine levels and side-effect severity were evaluated using Spearman’s r. Frequencies of cytokine-producing or tetramer-positive T cells and phagocytic scores were compared using non-parametric tests. All tests were two-tailed; p < 0.05 was considered significant.

## Supplementary Material

Supplementary Files

This is a list of supplementary files associated with this preprint. Click to download.

- SupplementaryTables.xlsx
- supplementaryfigs.pdf

**Supplementary Figure 1 | Heatmap of the covariation matrix for all observed side effects in the discovery cohort (Self-identified White, N = 50,535).** Cells display correlation coefficients ranging from −0.5 to 1, with positive values indicating that symptoms tend to occur together more frequently, 0 showing no co-occurrence and negative values indicating inverse relationships. Rows and columns represent individual side effects; color red (strong correlation) to blue (weak correlation) reflects the magnitude of the correlation.

Supplementary Figure 2 | Meta-analysis of the association between HLA-A*03:01 and vaccine reactogenicity across ancestry groups in discovery cohort [N = 57,938]. The analysis included White (N = 50,535), Black (N = 940), Hispanic (N = 4,287), and Asian or Pacific Islander (N = 2,176) cohorts. Points indicate odds ratios (ORs) and horizontal lines represent 95% confidence intervals; box sizes are proportional to inverse-variance weights. The vertical solid line marks the null value (OR = 1). The diamond represents the pooled estimate under a common-effect model. Models were adjusted for age and sex.

Supplementary Figure 3 | Forest plot showing association between HLA-A*03:01 and vaccine side effects by self-reported ancestry in discovery cohort [N = 57,938]. White [N= 50535] shown in blue, Black [N= 940] shown in orange, Hispanic [N=4287] shown in green and Asian or Pacific Islander [N= 2176] shown in red. Points indicate odds ratios and horizontal lines represent 95% confidence intervals; the vertical dashed line marks the null value (OR = 1), adjusted for age and sex.

**Supplementary Figure 4 | Proportion of vaccinated individuals who experienced three or more systemic side effects vs. two or fewer side effects.** Bar plot showing the proportion of individuals who experienced side effects (three or more systemic side effects vs. two or fewer side effects; colored in red and blue respectively) from each of three COVID-19 vaccine brands [Pfizer vaccinated (N = 26,250), Moderna vaccinated (N = 18,307), and Johnson & Johnson vaccinated (N = 3,312)].

Supplementary Figure 5 | Anti-Spike antibody positivity rate by 0, 1, and 2 copies of HLA-A*03:01. Violin plots showing the distribution of antibody positivity rates (AU/mL) stratified by HLA-A*03:01 allele copy number (0, 1, and 2 copies) in an independent cohort of 156 individuals (European ancestry). Inner boxplots indicate the median and interquartile range (IQR), with whiskers representing the range of the data.

**Supplementary Figure 6 | Gating strategy for identification and functional assessment of KCY-specific CD8**^**+**^ **T cells.** (**A**) PBMC or peptide-specific CD8^+^ T cell lines gating strategy. (**B**) CD8^+^ T cells were single cell sorted based on their tetramer specificity or (**C**) were assessed by function in an ICS assay. (**B**) Schematic displaying representative tetramer staining of HLA-A*03:01-KCY tetramer^+^ cells. (**C**) Representative gating for the assessment of functionality from an ICS assay.

**Supplementary Figure 7 | Modest Spike-specific CD4**^**+**^ T cell responses after COVID-19 vaccination in HLA-A*03:01^**+**^ **samples.** Cell lines generated by stimulating PBMCs with the Spike peptide pool and restimulated with the Spike peptide pool. (**A-E**) Frequencies of IFNγ^+^, TNF^+^, IL-2^+^, MIP-1β^+^ and CD107a^+^ CD4^+^ T cells from HLA-A*03:01^+^ samples before and after vaccination (V0: n = 9, V1: n = 12, V2: n = 12). Bars show mean – SD and symbols denote individual donors. Data represent biological replicates from independent donors (n) with one technical replicate per condition. Statistical comparisons were performed using a two-sided Mann–Whitney U test and were all not significant.

**Supplementary Figure 8 | Spike- and KCY-specific CD8**^**+**^ **T cell responses before COVID-19 vaccination.** Flow cytometry plots showing the frequencies of IFNγ^+^ CD8^+^ T cells following re-stimulation with either the Spike-derived peptide pool (Spike pool) or the KCY peptide (KCY) compared with negative control (NC) at baseline in HLA-A*03:01^+^ samples (V0, n = 9). Data represent biological replicates from independent donors (n) with one technical replicate per condition.

**Supplementary Figure 9 | Spike- and KCY-specific CD8**^**+**^ **T cell responses after one dose of COVID-19 vaccination.** Flow cytometry plots showing the frequencies of IFNγ^+^ CD8^+^ T cells following re-stimulation with either the Spike-derived peptide pool (Spike pool) or the KCY peptide (KCY) compared with negative control (NC) after one vaccine dose in HLA-A*03:01^+^ samples (V1, n = 12). Data represent biological replicates from independent donors (n) with one technical replicate per condition.

**Supplementary Figure 10 | Spike- and KCY-specific CD8**^**+**^ **T cell responses after two doses of COVID-19 vaccination.** Flow cytometry plots showing the frequencies of IFNγ^+^ CD8^+^ T cells following re-stimulation with either the Spike-derived peptide pool (Spike pool) or the KCY peptide (KCY) compared with negative control (NC) after two vaccine doses in HLA-A*03:01^+^ samples (V2, n = 12). Data represent biological replicates from independent donors (n) with one technical replicate per condition.

**Supplementary Figure 11 | Spike-specific CD4**^**+**^ **T cell responses before and after one dose of COVID-19 vaccination.** Flow cytometry plots showing the frequencies of IFNγ^+^ CD4^+^ T cells following re-stimulation with the Spike-derived peptide pool (Spike pool) compared with negative control (NC) at baseline (V0, n = 9) and after one (V1, n = 12) vaccine dose in HLA-A*03:01^+^ samples. Data represent biological replicates from independent donors (n) with one technical replicate per condition.

**Supplementary Figure 12 | Spike-specific CD4**^**+**^ **T cell responses after two doses of COVID-19 vaccination.** Flow cytometry plots showing the frequencies of IFNγ^+^ CD4^+^ T cells following re-stimulation with the Spike-derived peptide pool (Spike pool) compared with negative control (NC) after two vaccine doses in HLA-A*03:01^+^ samples (V2, n =10). Data represent biological replicates from independent donors (n) with one technical replicate per condition.

**Supplementary Figure 13 | Modest Spike-specific CD8**^**+**^ **T cell responses compared with Flu-NP**_**265**_ in HLA-A*03:01^**+**^ **samples.** (**A**) Flow cytometry plots of IFNγ^+^ CD8^+^ T cells following stimulation with the HLA-A*03:01-restricted Flu-NP_265_ peptide (n = 6). Non stimulated cell (NC: negative control) plots on the left and plots upon Flu-NP_265_ peptide stimulation on the right. (**B-D**) Paired comparison of KCY- and influenza NP_265_-specific responses showing IFNγ (**B**), TNF (**C**) and CD107a (**D**) production by CD8^+^ T cells from HLA-A*03:01^+^ samples (n = 6). Bars represent mean – SD; symbols denote individual donors. V1, post-vaccination dose 1; V2, post-vaccination dose 2. Data represent biological replicates from independent donors (n) with one technical replicate per condition.

Supplementary Figure 14 | *Ex vivo* tetramer-associated magnetic enrichment (TAME) plots of HLA-A*03:01-KCY-specific CD8^**+**^ **T cells.** *Ex vivo* tetramer-associated magnetic enrichment (TAME) plots of HLA-A*03:01-KCY-specific CD8^+^ T cells across before (V0, n = 4) and after vaccination (V1, V2, n = 4, respectively). Unenriched: fraction before tetramer magnetic enrichment; Flow-through depleted fraction after tetramer magnetic enrichment; TAME: tetramer magnetic enriched cells. Data represent biological replicates from independent donors (n) with one technical replicate per condition.

**Supplementary Figure 15 | Phenotypic analysis of tetramer**^**low**^ **and tetramer**^**high**^ HLA-A*03:01-KCY^**+**^ **T cells.** (**A**) Phenotype of KCY^+^ T cells *ex vivo* showing naïve (CD45RA^+^CCR7^+^CD95-), T_EM_ (CD45RA-CCR7-), T_CM_ (CD45RA-CCR7^+^), T_SCM_ (CD45RA^+^CCR7^+^CD95^+^), and T_EMRA_ cells (CD45RA^+^CCR7-) for HLA-A*03:01^+^ samples (n = 4) at V0, V1 and V2. The low-MFI tetramer^+^ T cells are represented as red dots and the high-MFI in blue. (**B**) HLA-A*03:01-KCY^+^ T cells phenotype in samples > 400 days post-third vaccine dose (vacSG64-V5, vacSG86-V5). Data represent biological replicates from independent donors (n) with one technical replicate per condition.

Supplementary Figure 16 | Phenotypic analysis of HLA-A*02:01-S_**269**_^**+**^ **T cells.** (**A**) *Ex vivo* tetramer-associated magnetic enrichment and staining of CD8^+^ T cells with HLA-A*02:01-restricted Spike-derived S_269_ tetramer with PBMCs collected two weeks after vaccination dose 1 (V1) and 2 (V2) (n = 3, respectively). (**B**) Phenotype of HLA-A*02:01-S_269_^+^ T cells *ex vivo* showing naïve (CD45RA^+^CD27^+^CD95-), T_EM_ (CD45RA-CD27-), T_CM_ (CD45RA-CD27^+^), T_SCM_ (CD45RA^+^CD27^+^CD95^+^), and T_EMRA_ cells (CD45RA^+^CCR7-) for HLA-A*02:01^+^ samples (n = 3) at V1 and V2 as pink dots. (**C**) HLA-A*02:01-S_269_-specific T cells phenotype represented as naïve (red), effector memory (T_EM_, orange), central memory (T_CM_, black), stem like memory cells (T_SCM_, grey), and terminally exhausted memory re-expressing RA cells (T_EMRA_, blue) phenotype. Bars show mean – SD; symbols denote individual donors. Data represent biological replicates from independent donors (n) with one technical replicate per condition.

**Supplementary Figure 17 | Double tetramer staining reveals peptide specificity of KCY**^**+**^ **T cells *ex vivo*.** *Ex vivo* tetramer-associated magnetic enrichment of CD8^+^ T cells with the HLA-A*03:01-restricted Spike-derived KCY tetramer (PE) and staining with HLA-A*03:01-restricted Influenza-derived NP_265_ tetramer (APC).

Supplementary Figure 18 | Cytokine responses to Spike, Spike-derived peptide pool, and LNP stimulation in HLA-A*03:01^**+**^ and HLA-A*03:01- samples. Cytokine concentrations measured in PBMC supernatants from HLA-A*03:01^+^ (n=27, dots) and HLA-A*03:01- (n=28, triangles) samples following stimulation with full-length Spike protein, Spike-derived peptide pool, or lipid nanoparticles (LNP). Data are shown for IL-2, IL-4, IL-5, IL-7, IFNa, IL-9, Eotaxin, IL-13, LT-a, IL-12p70, IP-10, GM-CSF, MCP-1, IL-1α and IFNγ. Data represent biological replicates from independent donors (n) with one technical replicate per condition. Statistical comparisons were performed using a two-sided Mann–Whitney U test.

**Supplementary Figure 19 | Gating strategy for identification of IL-6**^**+**^ **and IL-8**^**+**^ **cytokine-producing immune cell subsets following Spike stimulation.** Representative flow cytometry plots illustrating the gating strategy used to identify cytokine-producing subsets within Spike-stimulated PBMCs. PBMCs were first gated using a wider FSC-A/SSC-A gate designed to include lymphocytes as well as monocytes, B cells, and NK cells, rather than restricting the gate to lymphocytes alone; followed by singlet discrimination and exclusion of dead or dump-channel–positive events. Major immune subsets were subsequently identified as CD3^+^ T cells, CD19^+^ B cells, CD56^+^ NK cells, and CD14^+^ monocytes. IL-6^+^ and IL-8^+^ events were then quantified within each subset. Plots show one representative donor from each gating hierarchy. Gating was performed in FlowJo v10.9.

**Supplementary Figure 20 | Monocyte activation and Spike uptake.** (**A**-**B**) Indicative flow cytometry plots of CD14^+^ cells from four HLA-A*03:01^+^ PBMC samples (**A**) before and (**B**) after stimulation with the full-length Spike protein. (**C**) Representative confocal microscopy images of PBMCs from an HLA-A*03:01^+^ sample showing uptake of Spike protein–coated fluorescent microbeads (green, Alexa-488) and co-staining with Alexa-555 phalloidin (red) and DAPI (blue).

Supplementary Figure 21 | Level of cytokines in HLA-A*03:01^**+**^ **samples before and after one or two vaccine doses.** Cytokine levels measured in sera from HLA-A*03:01^+^ samples (n = 7) for IL-6, IL-8, TNF and IFNg collected before vaccination (V0, brown dots), two weeks post-vaccination after the first dose (V1, red dots) and two weeks post 2^nd^ dose of vaccination (V2, pink dots). Bars represent mean – SD; symbols denote individual donors. Data represent biological replicates from independent donors (n) with one technical replicate per condition. Pairwise comparisons between time points were performed using Wilcoxon matched-pairs signed-rank tests with correction for multiple comparisons.
